# An investigation of motor learning during side-step cutting, design of a randomised controlled trial

**DOI:** 10.1186/1471-2474-11-235

**Published:** 2010-10-13

**Authors:** Anne Benjaminse, Koen APM Lemmink, Ron L Diercks, Bert Otten

**Affiliations:** 1 University Medical Center Groningen, Faculty of Medical Sciences, Center for Human Movement Sciences, University of Groningen, Antonius Deusinglaan 1, 9713 AV Groningen, The Netherlands; 2 Hanze University Groningen, University of Applied Sciences, School of Sports Studies, SportsFieldLab Groningen, Zernikeplein 11, 9747 AS, Groningen, The Netherlands; 3 Department of Orthopaedic Surgery, University Medical Center Groningen, University of Groningen, PO Box 30.001, 9700 RB, Groningen, The Netherlands

## Abstract

**Background:**

Of all athletic knee injuries an anterior cruciate ligament (ACL) rupture results in the longest time loss from sport. Regardless of the therapy chosen, conservative or reconstructive, athletes are often forced to reduce their level of physical activity and their involvement in sport. Moreover, a recent review reported prevalences of osteoarthritis ranging from 0% to 13% for patients with isolated ACL-deficient (ACL-D) knees and respectively 21% to 48% in patients with combined injuries. The need for ACL injury prevention is clear. The identification of risk factors and the development of prevention strategies may therefore have widespread health and economic implications. The focus of this investigation is to assess the role of implicit and explicit motor learning in optimising the performance of a side-step-cutting task.

**Methods/design:**

A randomized controlled laboratory study will be conducted. Healthy basketball players, females and males, 18 years and older, with no previous lower extremity injuries, playing at the highest recreational level will be included. Subjects will receive a dynamic feedback intervention. Kinematic and kinetic data of the hip, knee and ankle and EMG activity of the quadriceps, hamstrings and gastrocnemius will be recorded.

**Discussion:**

Female athletes have a significantly higher risk of sustaining an ACL injury than male athletes. Poor biomechanical and neuromuscular control of the lower limb is suggested to be a primary risk factor of an ACL injury mechanism in females. This randomized controlled trial has been designed to investigate whether individual feedback on task performance appears to be an effective intervention method. Results and principles found in this study will be applied to future ACL injury prevention programs, which should maybe more focus on individual injury predisposition.

**Trial registration:**

Trial registration number NTR2250.

## Background

Of all athletic knee injuries an anterior cruciate ligament (ACL) rupture results in the longest time loss from sport [1]. Regardless of the therapy chosen, conservative or reconstructive, athletes are often forced to reduce their level of physical activity and their involvement in sport [2]. Moreover, a recent review reported prevalences of osteoarthritis ranging from 0% to 13% for patients with isolated ACL injury and 21% to 48% in patients with combined injuries [3], indicating additional long-term medical expenses [4]. The identification of risk factors and the development of prevention strategies may therefore have widespread health and economic implications.

The majority of research into noncontact ACL injury risk factors and the accompanying gender disparity has focused on the neuromuscular and biomechanical risk factors because of their potential for modification. Neuromuscular training strategies focussing on warm-up, technique, balance, strengthening and agility exercises have continued to evolve and represent an ever-increasing and equally important research focus [5-11]. Recent epidemiological data, however, suggest that in spite of these ongoing initiatives and reported early successes [5,8,12,13], ACL injury rates and the associated gender disparity have not diminished [14-16]. The disparity between positive laboratory results and actual effects on injury outcomes in high-risk female populations suggests a missing link between current research and clinical applications for neuromuscular training interventions [17]. One problem could be the difficulties with the measurements of injury rates and the difficulties with the implementation of thorough large scale injury prevention programs. Another issue could be the fact that the transition from conscious awareness during training sessions to unexpected and automatic movements during a training or game involves complicated motor control adaptations. Post-intervention lower extremity positions in the laboratory do not necessary reflect those on the field. The transition from conscious awareness during training sessions on technique in the laboratory to unexpected and automatic movements during a training or game involves complicated motor control adaptation. The purpose of this research project is to highlight the issue of motor learning in optimising sports performance in a manner consistent with ACL injury prevention.

Instructions can be an effective means of conveying goal-related information and educators commonly use them to teach and refine motor performance at all levels of skill [18]. During our intervention we will use the concept of implicit and explicit learning. Implicit motor learning refers to the acquisition of a motor skill without the concurrent acquisition of explicit knowledge about the performance of a skill that is normally processed in an automatic way, while explicit motor learning does refer to acquiring motor skills with an internal focus and specific knowledge about the performance of a skill [19]. The performance and learning of motor skills has been shown to be enhanced if the performer adopts an external focus of attention (focus on the movement effect) compared to an internal focus (focus on the movements themselves) [20].

There are ACL injury prevention programs addressing explicit rules regarding desired landing positions by emphasizing proper alignment of the hip, knee, and ankle [8-12,21-28]. However, the use of explicit strategies may be unsuitable for the control of complex motor skills [19]. It has been shown, that instructions that direct performers' attention to his or her own movements can actually have a detrimental effect on performance and learning and disrupt the execution of automatic skills, particularly in comparison with an externally directed attentional focus [20,29-32]. The exact reasons for the beneficial effects of an external focus of attention are still relatively unclear. However, trying to consciously control one's movements might interfere with the normal, automatic motor control processes, leading to a breakdown in the natural coordination of the movement [32,33]. Motor skills that are acquired explicitly tend to be less resilient under psychological [33-37] and physiological pressure [38,39], tend to interfere with the normal automatic processing of the motor schema [33,40], tend to be less durable [41] and less robust [42] when a fast response is required and explicit learning may be affected to a greater extent by an individual's intelligence than implicit learning [43-45]. Taking these benefits into account, implicit learning should be made more appealing in modern sporting arenas, since motor skills are often performed in anxiety-provoking competitions, under fatigued conditions. Having to consciously control a movement is considered to be a great disadvantage for athletes where attention to the game, players and ball and fast acting is required and thus very little or no attention could be given to a most optimal lower extremity position. A high-cognitive task will be vulnerable during a game.

In the ACL injury enigma in particular, psychological and physiological pressure is an important factor. Myklebust et al. reported that athletes are at a higher risk of suffering an ACL injury during a game than during practice [9]. Fatigue has also been proposed to be a contributor to non-contact ACL injuries [46-48]. For obvious reasons, a game constitutes more psychological and physiological stress compared to a practice session. Especially in later stages of competition, fatigue may have a cumulative, unfavorable effect on neuromuscular control and may potentially result in hazardous movement strategies [49]. The decreased capacity for controlling body movements after fatigue will potentially be more prominent when appropriate landing techniques have been taught in an explicit manner. Also, the possibility that implicit learning may immunize the athlete against the often debilitating influence of psychological stress on motor output should not be ignored.

Considering the benefits of implicit learning mentioned above, we feel that in the prevention of ACL injuries, we need to discover the possibilities of implicit learning. We use visual feedback (ie. observational learning) during our trial, where imitation of what is shown plays an important role. Imitation is the copying of body movements that is observed [50]. The human mirror system forms the foundation of observational learning [51]. Mirror neurons mediate understanding of action because neurons that represent an action are activated in the observer's premotor cortex. This automatically induced, motor representation of the observed action corresponds to that which is spontaneously generated during active action and whose outcome is known to the acting individual. Mirror neurons are visuomotor neurons that fire both when an action is performed and when a similar or identical action is passively observed [52]. A template of the movement becomes active through the mirror neurons by which the movement itself becomes clear in terms of motor actions, without high cognitive reflections [53]. This automatically induced, motor representation of the observed action corresponds to that which is spontaneously generated during active action and whose outcome is known to the acting individual. An important functional aspect of mirror neurons is therefore the relation between their ability to link visual and motor properties.

When observers see a motor event that shares features with a similar motor event present in their motor repertoire, they are primed to repeat it. The greater the similarity between the observed event and the motor event, the stronger the priming is [54]. The activation of motor representations through mere observation could have important applications in enhancing skill learning and in motor rehabilitation [55]. We therefore want to apply an intervention in which the subjects see their own performance, implicitly or explicitly. Priming will be strong, as seeing one's own performance will result in great similarity between the observed event and the executed event.

The goal of this research project is to investigate how we can train athletes individually to use certain motor patterns, that eventually become automatic. Motor learning that is offered in an implicit manner, will potentially be more robust once on the field. Our research project might give more insight in the ongoing problem of ACL injuries and might give the opportunity to more effectively implement prevention programs targeted towards the individual needs. If individual visual implicit feedback on task performance appears to be an effective intervention method, this could be applied to larger populations participating in team sports with a high risk of sustaining an ACL injury. Results and principles found in this study will be applied to future ACL injury prevention programs.

## Methods/Design

### Study design

This will be a randomised controlled laboratory study with two between-subject factors (gender and intervention group (ie. implicit, explicit and control group)) and one within-subject factor (time, ie. pretest, one week posttest and one month posttest). After consent to participate, subjects will be randomly assigned to a group based on the order in which they sign up for the initial baseline testing session. As an equal number of males and females per group (2 × intervention group and 1 × control group) is essential, stratified sampling will be carried out. The study design, procedures and informed consent are approved by the local Medical Ethical Committee (registration number 2009-142).

### Study population

Because the magnitude of the gender discrepancy in ACL injury rate is not consistent across sports [56-58], it is essential to study a specific group of athletes. As basketball is a high risk sport in terms of ACL injury [14,56,59], basketball athletes will be investigated in this study. Basketball players will serve as the control group, which will be an age- and activity level-matched will be included for this study. Inclusion criteria are: 18 years or older, playing basketball at the highest recreational level, no history of major lower extremity injury or surgery, no current or recent (6 months) injury to the entire lower extremity and able to participate in training and games for 100% at time of testing. Subjects will be excluded if they have had: any hip, knee or other relevant injury in the last 6 months prior to testing, any relevant previous injury or surgery at any joint of the lower extremity or any history of neurological, vestibular or visual impairment. Potential subjects will be recruited from regional basketball clubs and schools. Subjects will contact the primary investigator to schedule the testing session. Prior to testing, the subjects will be screened by the physical therapist (A.B.) on ACL status (ie. lesion, partial lesion, no lesion) The Lachman test and pivot shift test will be performed. The Lachman test has a very high sensitivity (85%) and specificity (94%). The pivot shift test is very specific, namely 98% [60]. In case of a lesion or partial lesion of the ACL, subjects will be excluded from the study. Furthermore, subjects may terminate participation under each circumstance.

We take the knee abduction moment and the loading rate of knee abduction moment over time as the main variables of interest as the main training purpose of this study is to get alignment of the leg in line with the GRF. When taking the clinical relevance of an 'at risk' and a 'not at risk' abduction moment into account, we refer to the prospective study by Hewett et al. [61]. The females in that study who ruptured their ACL (n = 9) had a greater stance phase peak external knee abduction moment, -45.3 ± 28.5 Nm, compared to that of uninjured females (n = 390), -18.4 ± 15.6 Nm (P < 0.001). We considered this mean difference in knee abduction moment to be clinically relevant, as it was predictive of ACL injury occurrence in a prospective study. We therefore used the difference to calculate the power for this study. With an effect size of 0.55 (determined based on those differences of the means divided by the pooled SD) and an alpha of 0.05, we reach a power of 0.80 when including 120 subjects, which means 40 subjects (20 females and 20 males) per group (ie. implicit learning group, explicit learning group and control group). G*Power for Mac, Version 3.1.2 has been used to calculate the needed sample size.

### Intervention

Two types of immediate visual feedback will be given (Table 1):

**Table 1:** Testing schedule

	Explicit Learning Group (n = 40)	Implicit Learning Group (n = 40)	Control Group (n = 40)
T1 Intervention	30 side-step cutting trials - Detailed verbal instructions on performance will be given	30 side-step cutting trials - Best performance will be shown	30 side-step cutting trials - No feedback on performance will be given
T2 Retention test (one week post intervention)	30 side-step cutting trials - No feedback on performance will be given	30 side-step cutting trials - No feedback on performance will be given	30 side-step cutting trials - No feedback on performance will be given
T3 Retention test (one month post intervention)	30 side-step cutting trials - No feedback on performance will be given	30 side-step cutting trials - No feedback on performance will be given	30 side-step cutting trials - No feedback on performance will be given

1) Explicit feedback: After each sidestep cutting manoeuvre, subjects will immediately receive explicit instructions to improve their performance. Potential ACL injury risk factors include: 1) Increased knee valgus angle [61], 2) Decreased knee flexion angle [62-64], 3) Increased anterior tibial shear force [65,66], 4) Decreased hip flexion angle [62,64,67,68], 5) Increased hip internal rotation angle [62], 6) Increased knee internal rotation angle [69]. Items to improve these potential risk factors above will be mentioned to the subjects and subjects will be requested to minimise the load at the knee.

2) Implicit feedback: Subjects will undergo a dynamic visual feedback intervention. Each time after a subject has performed the task, a visual representation of the best performance so far of the whole body (3D posterior view) will be shown to the subject with the Basler recordings (Darwinian learning). No explicit feedback or instructions at all will be given, however subjects will know in advance that there are superior and inferior ways to perform the task. The subject will search by him-/herself for the solution that fits best in their body; they explore and then select the performance which fits best.

The best performance so far will be based on the peak valgus moment, which needs to be as low as possible. The total training session consists of 30 trials. As we need to make sure that the improvements in landing performance (if any) are permanent rather than temporary, a retention test will be conducted one week (similar to Onate et al.) [70] and one month later. During the retention tests no feedback at all will be given to either group. The control group will perform the exact same tasks as the intervention groups. The control group, however, will not receive any feedback during any trial. To make sure three homogeneous groups are participating, five trials with no feedback will be performed prior to the real intervention (ie. in addition to the 30 trials).

### Measurements

Screening for inclusion and exclusion criteria will occur at the laboratory setting and will be performed by the primary investigator (A.B.) of this study. All subjects will sign an informed consent form in accordance with the University of Groningen Medical Ethics Committee prior to participation.

Lower extremity kinematic, kinetic and EMG data of subjects performing a sidestep-cutting manoeuvre in the laboratory will be analysed. The primary outcome measurements will be as follows:

1) Knee abduction moment

2) Loading rate of knee abduction moment over time

Secondary outcome parameters will be as follows:

3) Average EMG pattern of the gluteus maximus (GM), vastus medialis (VM), vastus

lateralis (VL), medial hamstring (MH), lateral hamstring (LH), medial gastrocnemius

(MG) and lateral gastrocnemius (LG)

4) Muscle onset time (ie. the first burst in EMG as detected by the Santello algoritm prior

to landing [66]) of the GM, VM, VL, MH, LH, MG and LG

5) Muscle activity of the GM, VM, VL, MH, LH, MG and LG integrated over the interval from 100 milliseconds prior to foot contact to foot contact (preparatory interval) and from foot contact to the point of peak knee flexion (weight acceptance)

6) Muscle co-contraction (ie. using the integrated EMG of each muscle and the formula: [(less active muscle/more active muscle) X (sum of the integrated activity of both muscles)]) of VL-MG, VL-LH, VM-LG and VM-MH over the interval from 100 milliseconds prior to foot contact to foot contact (preparatory interval) and from foot contact to the point of peak knee flexion (weight acceptance)

7) Hip, knee and ankle angles at IC, peak posterior GRF and the maximum values for each of those variables

a. Abduction/adduction

b. Flexion/extension

c. External/internal rotation (only for hip and knee)

8) Knee angular displacement flexion angle

9) Joint moments of hip, knee and ankle at IC, peak posterior GRF and the maximum values for each of those variables

a. Abduction/adduction (not for knee, see primary outcome measurement)

b. Flexion/extension

c. External/internal rotation (only for hip and knee)

In addition we will record the history on injuries and/or surgeries, the Tegner activity level questionnaire [69,70] and activity between intervention and follow up tests.

### Subject preparation

In order to calculate the hip, knee and ankle joint angles and moments, anthropometric measurements will first be taken in preparation of the 3D motion analysis testing. Anthropometric measurements will include body weight and height, knee and ankle diameter and leg length (ASIS - medial malleolus). Subjects will then have reflective markers placed over the heel, lateral malleolus, second metatarsal head, femoral epicondyle and ASIS and PSIS bilateral. Another four markers will be placed bilaterally on the lateral side of the mid-thigh and mid-calf. EMG signals will be recorded using silver-silver chloride, pre-gelled bipolar surface electrodes (ZeroWire EMG, Aurion, Italy). Electrode locations will be located via palpation of the subject's anatomy and will be placed over the appropriate muscle belly in line with the direction of the fibres with an interelectrode distance of approximately 20 mm, which is in accordance with the work of Delagi and Perotto [71]. Electrode sites will be shaved, abraded and cleaned with isopropyl alcohol to reduce impedance. The electrodes will be secured to the subject's skin with tape to minimise motion artefact. Electrode placement will be confirmed through visual inspection of signals on the computer screen using Vicon Nexus Software (Version 1.6, Vicon Motion Systems, Inc., Centennial, CO) during standardised manual muscle testing [29]. Two seconds of maximal voluntary isometric contraction (MVIC) EMG signals will be collected from each muscle before data collection. These data will be processed and used for normalisation of the corresponding muscle's EMG activity during the dynamic task. The same investigator (A.B.) will perform all electrode and marker placements. All the subjects will wear tight-fitting shorts and their own indoor basketball shoes.

An unanticipated cutting task will be carried out in the laboratory. Each athlete will complete a practice session that includes several anticipated and unanticipated trials of each of the two tasks to familiarise his-/herself with the experimental setup as well as to reduce the effect of targeting the force platform. The athlete will randomly perform cutting trials. The straight run and cross-step are catch tasks so as to present the athletes with three options. Consequently, the cutting manoeuvre becomes an unanticipated task. Specifically, the cutting manoeuvre consists of an approach run, followed by a plant-and-cut manoeuvre at a 45° angle with the dominant foot on the force platform. The cutting direction will be to the right for left-footed subjects and to the left for right-footed subjects. Each angle will be measured from the centre of the force plate and the corresponding line will be marked (using tape) so that it can be clearly seen by the subjects. As for the straight ahead run, the subjects continue the approach run through the experimental setup, with a change in neither direction nor speed.

Two infrared timing gates will be used to ensure that the approach speed will be 4.5-5.5 m/s and 0.5 seconds before the subjects land on the force platform and make the cut, a 3-light guiding system will be used to randomly cue the subjects; one light will turn on, indicating the direction the subject should go. Each subject will be given 1 minute between trials to reduce the potential effects of fatigue. To make sure there is pressure on the performance, the exit speeds needs to be 4.0-5.0 m/s, measured by the infrared timing gaits, 5 meters beyond the force plat form.

Only successful trials will be kept. A cutting trial is deemed successful if the subjects approaches the force platform with the required speed, performs the manoeuvre with the target board illuminating a light, makes IC with the force platform and either runs straight ahead or changes direction at a 45° cut angle with the required exit speed. Subjects will be required to continue running after sidestep execution for 5 meters. Cutting trials during which the subject modifies his/her stride length (i.e., stutter-step) to make contact with the force platform will also be discarded. The stutter-step will be discarded as these trial will not be comparable with the other trials; the speed will in all likelihood decrease and will not be between 4.5-5.5 m/s anymore. Approach speed is based on previous studies [69,72].

### Instrumentation

Vicon's Nexus software (Version 1.6) of the Vicon Motion Analysis System (Vicon Motion Systems, Inc., Centennial, CO) will be used to collect and calculate the kinematic and kinetic data. GRF data will be collected at 1200 Hz by 2 force plates (Bertec Corporation, Columbus, Ohio) that are located within a custom-built flooring system in which the force plates are flush with the surrounding surface. Surface electromyographic (EMG) signals will be collected with the Noraxon Telemyo Telemetry EMG System (Noraxon USA Inc, Scottsdale, AZ) using ZeroWire. Signals will be passed from the electrodes to the transmission unit. After amplification, the telemetry signals will then be passed from the transmitter to the receiver for further amplification (overall gain of 2000) and filtered with a bandwidth filter (10 Hz low pass 500 Hz high pass Butterworth filter, common mode rejection ratio of 130 db). Signals from receiver will be collected with the data acquisition package Vicon Nexus Software (Version 1.6, Vicon Motion Systems, Inc., Centennial, CO). The analog signal from the EMG receiver will be converted to a digital signal via a DT3010/32 (32 channel, 24 bit) A/D board (Data Translation, Inc., Marlboro, MA). Basler camera's (640 × 480, 210 fps, Vicon Motion Systems, Inc., Centennial, CO) with a 25 mm C-mount lens will be used to collect analog high-speed data.

### Data acquisition and statistical analysis

Hip, knee and ankle joint kinematics will be evaluated at IC, peak posterior GRF and the maximum values for each of those variables. IC will be defined as the point in time when 5% of the subject's body weight is upon the force plate. The raw coordinate data will be filtered using an optimised cut off frequency.

Raw analog data from the MVICs and synchronised raw analog data (joint kinematic, joint kinetic data and GRF data) from the jump trials will be imported into Matlab (release 12, The MathWorks, Natick, MA) for data processing and identification of the variables of interest. The mean value of each MVIC will be used for normalisation of the EMG during the jump trials. Both the MVIC and trial EMG data will be processed with a linear envelope before filtering by a Butterworth filter (fourth-order, zero-phase shift, cut off frequency of 20 Hz).

Raw analog data from the force plates will be used to calculate the GRF data for each jump trial. The raw coordinate data will be filtered using an optimised cut off frequency. The GRF will be filtered using a fourth-order Butterworth filter at a cut off frequency of 100 Hz. The GRF data will be used to calculate the maximum posterior GRF during the initial-stance phase of the jump tasks.

Only the dominant leg will be analysed. In addition to the 30 trials during the intervention and retention tests, all subjects will perform five trials to begin with. These five baseline trials (without feedback) prior to the intervention will be conducted to assure homogeneity across groups on the primary outcome measures (knee abduction moment and the loading rate of knee abduction moment over time). The results of these five trials will be compared to each other using a one-way ANOVA. Multilevel analysis will be applied to examine the within and between subject effects. The learning curve with implicit versus explicit versus no feedback of the primary outcome measures over time will be analysed using SPSS 18.0 (SPSS Inc., Chicago, IL). The secondary outcome measurements will be used as explanatory variables. In addition, post-hoc Bonferroni adjustments will be conducted for the within, between and interaction effects. An alpha level of 0.05 will be set a priori.

## Discussion

Female athletes have a significantly higher risk of sustaining an anterior cruciate ligament (ACL) injury than male athletes. The identification of risk factors and the development of prevention strategies may have widespread health and economic implications. Poor biomechanical and neuromuscular control of the lower limb is suggested to be a primary risk factor of an ACL injury mechanism in females [61]. These are modifiable characteristics, which may potentially reduce the ACL injury rate after proper intervention. But even though a lot of effort is put in the prevention of noncontact ACL injuries, the incidence remains high [14,15,56]. The purpose of this research project is to highlight the issue of motor learning in optimising sports performance in a manner consistent with ACL injury prevention. If individual visual feedback on task performance appears to be an effective intervention method, this could be applied to larger populations participating in team sports with a high risk of sustaining an ACL injury. Results and principles found in this study will be applied to future ACL injury prevention programs, which should maybe more focus on individual injury predisposition.

## Competing interests

The authors declare that they have no competing interests.

## Authors' contributions

AB wrote the manuscript, designed the study, coordinates the trial, will collect, analyse and report the data. BO will assist in analysing and reporting the data. BO, KAPML and RLD designed the study. All authors read, edited and approved the final manuscript.

## Pre-publication history

The pre-publication history for this paper can be accessed here:

http://www.biomedcentral.com/1471-2474/11/235/prepub
